# Cancer-Specific Biomarker hNQO1-Activatable Fluorescent Probe for Imaging Cancer Cells In Vitro and In Vivo

**DOI:** 10.3390/cancers10120470

**Published:** 2018-11-28

**Authors:** Surendra Reddy Punganuru, Hanumantha Rao Madala, Viswanath Arutla, Kalkunte S. Srivenugopal

**Affiliations:** Department of Pharmaceutical Sciences, School of Pharmacy, Texas Tech University Health Sciences Center, Amarillo, TX 79106, USA; hmadala@mgh.harvard.edu (H.R.M.); viswanath.arutla@ttuhsc.edu (V.A.)

**Keywords:** fluorescent probe, cancer biomarker, tumor diagnosis, cancer imaging, NAD(P)H quinone oxidoreductase 1, large Stokes shift, dicyanoisophorone

## Abstract

Human NAD(P)H quinone oxidoreductase-1 (hNQO1) is an important cancer-related biomarker, which shows significant overexpression in malignant cells. Developing an effective method for detecting NQO1 activity with high sensitivity and selectivity in tumors holds a great potential for cancer diagnosis, treatment, and management. In the present study, we report a new dicyanoisophorone (DCP) based fluorescent probe (NQ-DCP) capable of monitoring hNQO1 activity in vitro and in vivo in both ratiometric and turn-on model. NQ-DCP was prepared by conjugating dicyanoisophorone fluoroprobe with hNQO1 activatable quinone propionic acid (QPA), which remain non-fluorescent until activation by tumor-specific hNQO1. NQ-DCP featured a large Stokes shift (145 nm), excellent biocompatibility, cell permeability, and selectivity towards hNQO1 allowed to differentiate cancer cells from healthy cells. We have successfully employed NQ-DCP to monitor non-invasive endogenous hNQO1 activity in brain tumor cells in vitro and in xenografted tumors developed in nude mice.

## 1. Introduction

Human NAD(P)H quinone oxidoreductase-1 (hNQO1), formerly referred to as DT diaphorase, is a cytosolic flavoenzyme that plays an essential role in cellular protection against endogenous quinones (e.g., the vitamin E α-tocopherol quinone, menadione, benzene quinones) [1]. It is a homodimer which binds quinones with the co-factor NADH or NADPH, leading to a reduction of the quinones in a two-electron transfer reaction [2]. In addition to its enzymatic activity, NQO1 has been found to be involved in other biological processes such as antioxidant activity and the stabilization of essential regulatory proteins under stress [3]. The enzyme hNQO1 is constitutively expressed at relatively low levels in various normal tissues but is frequently expressed at high levels in most solid tumors such as breast [4], lung [5,6], prostate [7], stomach [8], colon [9], pancreatic [10], brain [11], head, and neck cancer [12]. NQO1 is expressed in many human solid tumors at levels of 200-fold above that in normal tissue and its elevated activity has been closely associated with tumor progression, aggressiveness, resistance to chemotherapy, and poor prognosis [4,5,6,7,8,9,10,11,12]. As such, hNQO1 has been recognized as a potential biomarker of human malignancies, accurate detection of which can be highly desirable to improve diagnostic efficacy and predict drug responsiveness [13].

The reasons for NQO1 overexpression in cancers are still unclear, however, the elevated oxidative stress prevalent in most malignancies appears to be a link [14]. Like many phase 2 metabolic enzymes inducible by redox stress, the 5′ upstream region of the NQO1 gene harbors typical antioxidant response elements (AREs) [15] that associate with the NRF2 (Nuclear factor erythroid-derived 2-like 2) transcription factor, which in turn is stabilized through dissociation from Keap-1 in oxidative milieu [16].

In common with other malignancies that possess higher levels of oxidative stress, the malignant gliomas constitute a highly invasive, heterogeneous, complex, and fatal tumor type, the extent of which is not precisely identifiable by modern imaging techniques [17]. Despite all the current treatment modalities for malignant gliomas, surgical resection is associated with a moderate recurrence-free survival of patients [18]. Therefore, maximal removal of the tumor mass is the primary goal in the treatment of this tumor type. However, the ability of the unaided human eye to detect accurate boarders between cancer and normal tissues during surgery is limited. Recently, fluorescence molecular imaging has emerged as an important aid to assist the optically guided surgery because of its high sensitivity and non-radiation application [19,20]. Particularly, enzyme activated turn-on fluorescent probes hold great promise in accelerating clinical translation by significantly improving the target to background ratio (TBR) and, in turn, the sensitivity and contrast of fluorescence imaging [21,22]. In this context, it is necessary to identify a cancer-specific trait, a biomarker that is stable, dependable and not significantly shared with normal cells.

Previously, hNQO1 activated fluorescent probes were developed based on its property of quinone bio-reduction [23], where the fluorescent probe conjugated with a quinone-based NQO1 substrate as a trigger group, which holds the fluorophore in a quenched state (Table 1). Intense fluorescence of these probes occurs upon specific activation of the quinone moiety by hNQO1. The “trimethyl lock” containing quinone propionic acid (QPA) has been extensively used as an hNQO1-responsive trigger group because of its rapid and selective reduction by hNQO1 to afford hydroquinone analog, which underwent lactonization to release fluorescent probe. Consequently, McCarley group and others have developed several QPA-based fluorescent probes for detection and imaging of hNQO1 in cancer cells. The fluorophores used in these studies include rhodamines [24,25], napthalimides [26,27,28], Acedan [29], coumarin [30], HO-BODIPY [31], tetraphenylethane [32], amino-acetyl-naphthalene [33], carbocyanines [34,35,36]. On the other hand, several affinity-based small molecule probes were also developed by linking the hNQO1 inhibitor as the recognition group [37,38]. Although these fluorescent probes have shown good selectivity even in the presence of other cellular reductants, their in vivo applications have been limited. Furthermore, the development of fluorescent probes with large Stokes shift is essential for biological applications to minimize the interference between the excitation source and the fluorescent emission along with the high target-to-background ratio. With these points in consideration, we developed a fluorescent probe for endogenous NQO1 imaging using a rationally developed fluorescence probe NQ-DCP with large Stokes shift. NQ-DCP displayed high sensitivity and selectivity against hNQO1 with excellent features such as low cytotoxicity, cell permeability and high target to background ratio in bioimaging NQO1 activity in brain tumors.

## 2. Results

### 2.1. Design of the hNQO1 Responsive Fluorescent Probe

**Figure 1:**
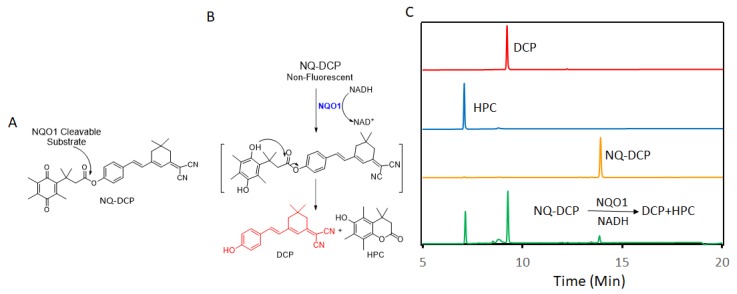
The NQ-DCP probe is specifically activated by NAD(P)H quinone oxidoreductase-1 (NQO1). (**A**) structure of NQ-DCP. (**B**) Mechanism of activation of non-fluorescent NQ-DCP in the presence of NADH/NQO1 and release of highly fluorescent dicyanoisophorone (DCP) (**C**) high-performance liquid chromatography (HPLC) analysis of NQ-DCP activation. HPLC spectra of NQ-DCP, DCP, dihydrocoumarin (HPC) and the HPLC spectrum of a reaction mixture containing 10 μM NQ-DCP, 2.5 μg/mL NQO1 and 100 μM NADH are shown.

A fluorogenic enzyme substrate probe NQ-DCP (Figure 1A) was rationally designed based on the “quinone trimethyl lock system” in which QPA is attached to a fluorophore dicyanoisophorone (DCP) via an ester bond. The synthesis of NQ-DCP was started from the preparation of the DCP according to the procedure outlined in Scheme S1 in the Supplementary Materials. The chemical structures of these compounds were characterized by ^1^H NMR, ^13^C NMR, and mass spectrometry. The ester bond with QPA reversibly quenches the fluorescence of the DCP; Upon redox “activation” by hNQO1, the quinone moiety is reduced to its corresponding *o*-hydroxydihydrocinnamic acid derivative that undergoes rapid lactonization under physiological conditions to yield dihydrocoumarin, 6-hydroxy-4,4,5,7,8-pentamethylchroman-2-one (HPC) along with a highly fluorescent DCP (Figure 1B). Activation of NQ-DCP in the presence of hNQO1 was monitored using HPLC analysis by incubating 10 μM NQ-DCP with purified hNQO1 (2.5 μg/mL) in the presence of 100 μM NADH for 30 min. The results indicated that reaction product exhibited chromatographic peaks at 9.2 min and 7.1 min, which matched perfectly with the DCP and HPC indicating the removal of QPA from the NQ-DCP. A weak peak was also observed at 13.8 min, corresponding to the unreacted NQ-DCP probe.

### 2.2. Spectroscopic Response of NQ-DCP to hNQO1

**Figure 2:**
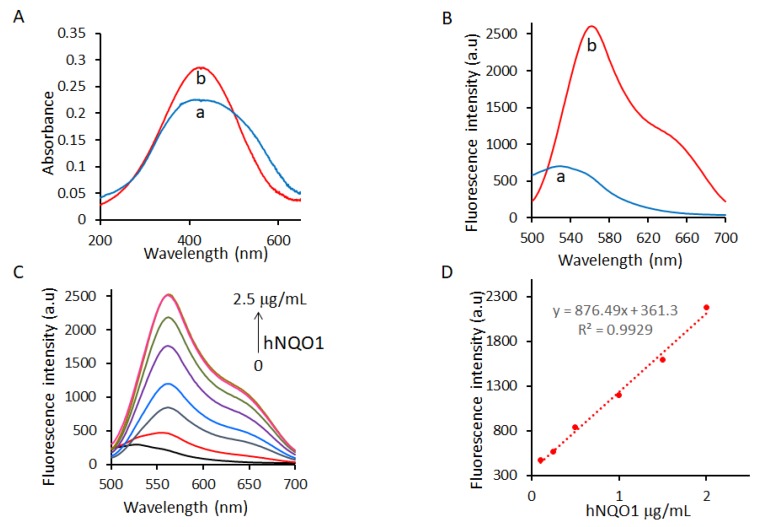
Changes in absorbance (**A**) and fluorescence (**B**) of NQ-DCP before(a) and after(b) activation by 2.5 μg/mL of hNQO1 in PBS with 0.1% BSA (pH 7.4). (**C**) Fluorescence response of NQ-DCP to different concentrations (0.12, 0.25, 0.5, 1.0, 1.5, 2.0 and 2.5 µg/mL) of hNQO1 in the presence of 100 µM NADH. (**D**) The linear fitting curve of fluorescence intensity towards the concentrations of hNQO1 from 0.12 to 2.5 µg/mL, λ_ex/em_ = 420/565 nm.

**Figure 3:**
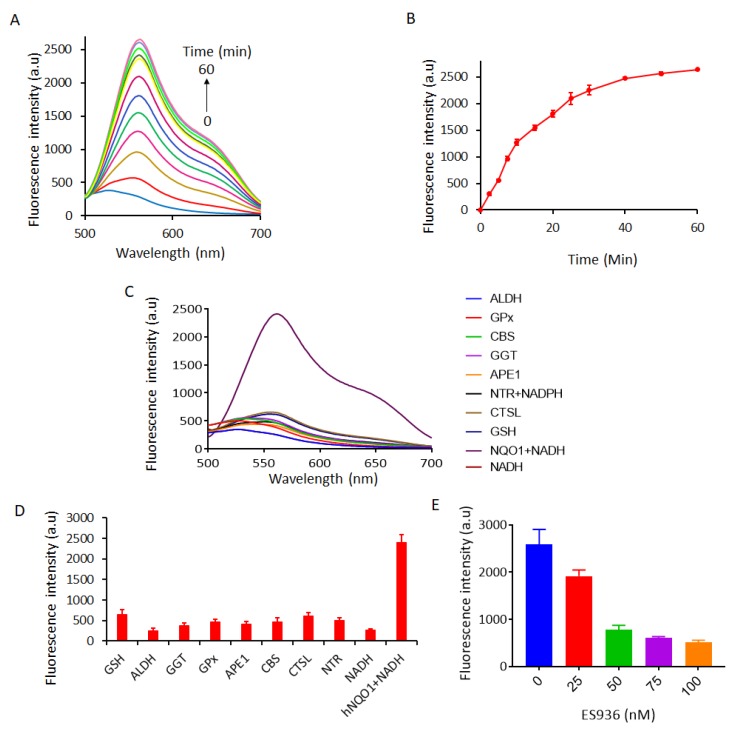
(**A**) Fluorescence spectrum of 10 µM NQ-DCP during a period of 60 min in the presence of 2.5 µg/mL of NQO1 and 100 μM of NADH. (**B**) Time-dependent fluorescent intensity changes for the probe NQ-DCI with different concentrations of NQO1 (0 to 25 µM) in PBS with 0.1% BSA (pH 7.4) at _λex/em_ = 460/646 nm. (**C**) Fluorescence response of NQ-DCP probe (10 µM) to various biologically relevant molecules/enzymes such as the GSH, ALDH, GGT, NTR, GPx, APE1, CBS, CTSL, NADH, GSTpi, NTR + NADH, NQO1, NQO1+NADH, in PBS containing 0.1% BSA (pH 7.4). Bars represent the average final fluorescence intensity at 565 nm after 30 min incubation in three independent experiments (**D**). (**E**) Inhibitory effect of ES936 on hNQO1 activation of NQ-DCP at different concentrations. The bar diagram represents mean fluorescence intensities (a.u.) ± SEM of NQ-DCP at 565 nm in the presence of different concentrations of ES936 (λ_ex/em_ = 420/565 nm).

The spectroscopic response of the NQ-DCP probe to an hNQO1 enzyme in the presence of NADH was evaluated in a physiologically relevant buffer (0.1% BSA and 100 μM NADH containing PBS) to determine its ability to detect hNQO1. The absorption and fluorescence spectra of NQ-DCP in the absence and presence of hNQO1 are shown in Figure 2A,B. In the presence of hNQO1, although there was a distinct color change from yellow to red there was no difference found in the absorption maximum (425 nm) of NQ-DCP (Figure 1A). Further, the reaction of NQ-DCP probe with hNQO1 produced a huge fluorescence enhancement at 565 nm with a large Stokes shift (140 nm) (Figure 2B), which is favorable for sensitive detection and bioimaging analysis, because of a non-overlapping fluorescence emission. Having demonstrated the response of NQ-DCP towards hNQO1, we then investigated its sensitivity for hNQO1 under optimal enzyme reaction conditions. We measured the fluorescence spectra of NQ-DCP (10 μm) following incubation with different concentrations of hNQO1 (0.12, 0.25, 0.5, 1.0, 1.5, 2.0, and 2.5 μg/mL (0.0038, 0.008, 0.016, 0.032, 0.048, 0.064, 0.08 μmol)) along with 100 μM NADH at pH 7.4 for 30 min. As shown in Figure 2C, in the absence of hNQO1, NQ-DCP was highly stable, with little or no fluorescence enhancement after 30 min. On incubation with hNQO1, the fluorescence intensity at 565 nm increased progressively with the NQO1 concentration and reached a maximum when the hNQO1 concentration was 2.5 μg/mL. The fluorescence intensity at 565 nm was linear with hNQO1 concentration, 0.25–2.0 μg/mL (Figure 2D). The hNQO1 detection limit (LOD) of NQ-DCP was calculated to be as low as 0.095 μg/mL, based on the standard deviation of the response (Sy) and the slope of the calibration curve (S) according to the formula: LOD = 3.3(Sy/S). Additionally, the fluorescence spectra showed a time-dependent trend (Figure 3A), with the emission peak at 565 nm increasing over time and reaching a maximum after 60 min (Figure 3B).

### 2.3. Selectivity of NQ-DCP Towards hNQO1

The specificity of NQ-DCP probe towards hNQO1 was studied by determining its reactivity towards various biomolecules such as the glutathione (GSH, 1 mM), aldehyde dehydrogenase 1 (ALDH1A1, 2.5 μg/mL), gamma-glutamyl transferase (GGT, 10 U), glutathione Peroxidase (GPx, 10U), apurinic/apyrimidinic endonuclease (APE1, 10U), cystathionine-β-synthase (CBS, 2 μg/mL), cathepsin L (CTSL, 2 μg/mL), glutathione S-transferase (GST-pi, 2 μg/mL), nitroreductase (NTR, 2 μg/mL) and NADH (100 μM) alone or the combination of and NQO1 (2.5 μg/mL). As shown in Figure 3C,D, NQ-DCP demonstrated a high selectivity for hNQO1 over the other enzymes/substrates tested. To gain more insight into the specificity of the probe towards hNQO1, the enzyme was pre-treated with ES936 (0–100 nM), a well-known hNQO1 inhibitor [39] and then incubated with NQ-DCP. As shown in Figure 3E, ES936 effectively suppressed the fluorescence response in a concentration-dependent manner, indicating the hNQO1-dependent fluorescence response.

### 2.4. Visualization of Cancer Cells by Fluorescence Imaging of hNQO1

**Figure 4:**
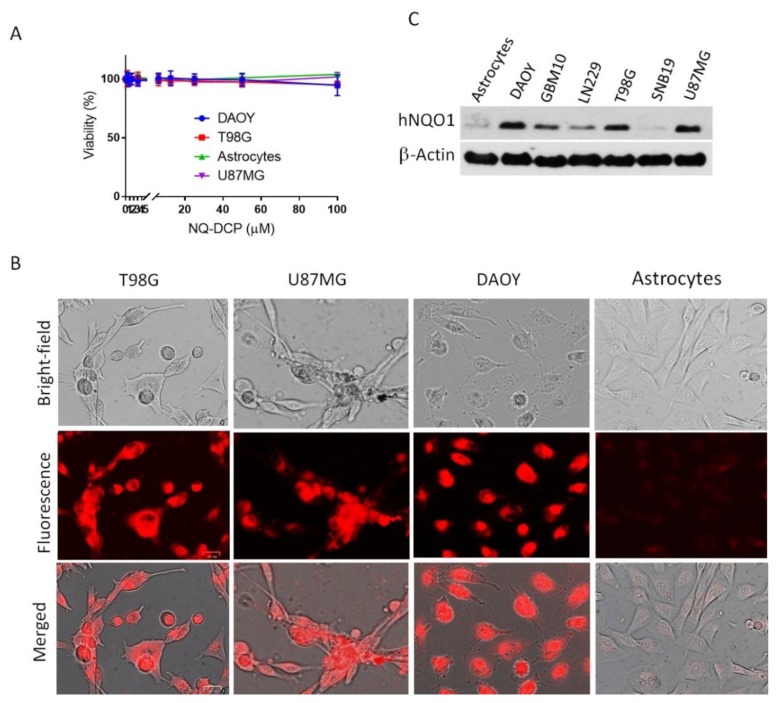
Comparison of NQO1 activity in human cancer cells (DAOY, T98G, U87MG) and normal cells (astrocytes) in response to NQO1 activated NQ-DCP probe. (**A**) Cytotoxicity of NQ-DCP against cancer cells (DAOY, T98G, and U87MG) and normal cells (astrocytes). Cell growth inhibition was analyzed by resazurin reduction assay. Cells were treated with 10 different dilutions of the NQ-DCP (100 μM to 0.196 μM) for 72 h. (**B**) Fluorescence images of NQO1-positive cancer cells and NQO1-negative normal cells after incubation of 10 µM NQ-DCP for 1 h. (**C**) Western blot showing the expression of NQO1 in cancer cells in comparison to the normal cells; β-actin served as a loading control.

Fluorescence microscopy was used to demonstrate the potential of NQ-DCP fluorescent probe in the visualization of NQO1 in human cancer cells. The cytotoxicity of NQ-DCP was initially evaluated in cultured brain tumor (DAOY, T98G, and U87MG) and normal (astrocytes) cells to assess its biocompatibility using the resazurin reduction assay [40]. The results revealed that NQ-DCP was nontoxic to both normal and cancer cells even at 100 µM after 72 h incubation (Figure 4A). With excellent biocompatibility demonstrated by the NQ-DCP, we investigated its performance in both cancers (DAOY, T98G, U87MG) and normal (astrocytes) cells. Cells were incubated with NQ-DCP (10 μM) for 60 min, washed with PBS and were imaged under a fluorescence microscope. The results are presented in Figure 4B. DAOY, T98G, and U87MG cells elicited bright fluorescence signals, whereas the normal cells failed to do so. The presence of hNQO1 protein in cancer cells and its absence in normal cells as determined by western blot analysis confirmed the probe reactivity in cells (Figure 4C), thereby indicating a good cell permeability of the NQ-DCP and its reaction with the intracellular hNQO1.

### 2.5. Applicability of NQ-DCP for Flow Cytometry

**Figure 5:**
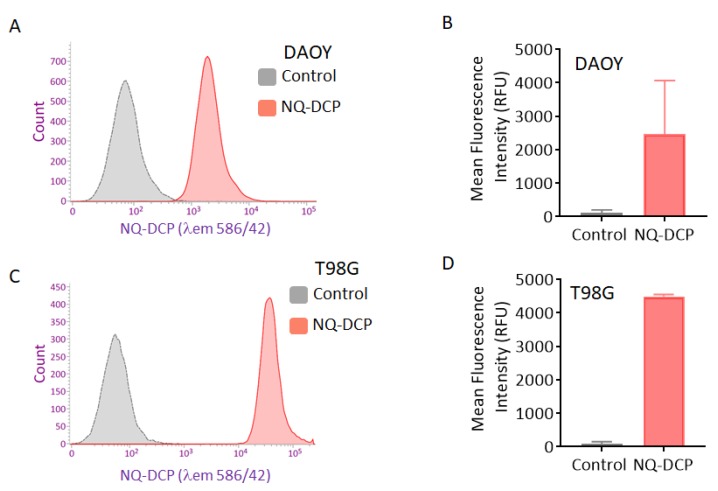
Flow cytometry assays of NQ-DCP activation by NQO1-positive cell lines (**A**) DAOY and (**C**) T98G. (**B**,**D**) Bar diagram represents the mean fluorescence intensities of three independent experiments. Assays were performed by counting 1 × 10^4^ cells that had been exposed to 10 µM NQ-DCP probe for 1 h; λ_em_ = 586/40 nm.

Flow cytometry assays were used to assess the applicability of the NQ-DCP probe to rapidly detect and quantify tumor cells containing hNQO1. The probe was incubated with cell suspensions (DAOY and T98G) for 30 min and a flowcytometry was used to measure the fluorescence (λ_em_ = 586/40) in about 1 × 10^4^ individual cells. The resulting histograms and mean fluorescence intensities for both DAOY and T98G cell lines are shown in Figure 5. A high-intensity unimodal distribution of signal was obtained for NQ-DCP activation in both NQO1-positive cancer cell lines. These results clearly demonstrated that NQ-DCP can quantitatively detect endogenous NQO1 and can be used to rapidly differentiate tumor cells in fluidic streams.

### 2.6. Evaluation of Specificity in Cancer Cells

**Figure 6:**
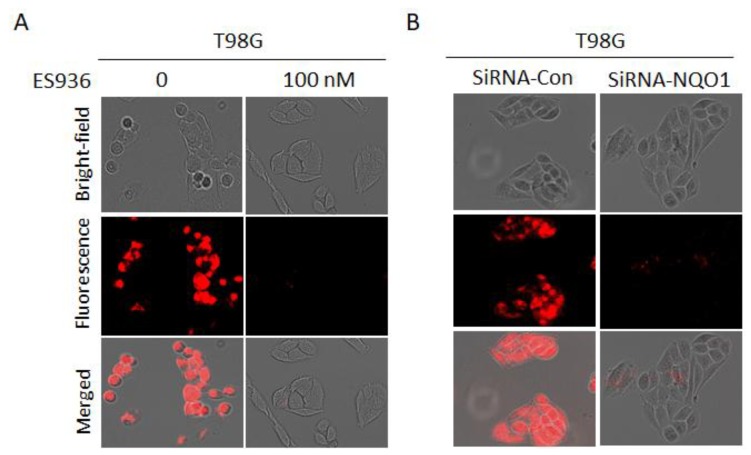
(**A**) Inhibitory effects of NQO1 inhibitor ES936 (100 nM) on NQ-DCI fluorescence. Fluorescence microscopy analysis of T98G cells after treating with NQ-DCP (10 μM) for 1h in the presence and absence of ES936 (100 nM). (**B**) The effect of small interfering RNA (siRNA) knockdown of NQO1 on the activation of NQ-DCP in T98G cells.

To show the substrate selectivity of NQ-DCP for hNQO1, T98G cells were pretreated with 100 nM of NQO1 inhibitor ES936 for 6h and incubated 10 μM of NQ-DCP. As shown in Figure 6A, ES936 completely blocked the NQ-DCP fluorescence when compared to the untreated cells. In addition, small interfering RNA (siRNA) silencing of the hNQO1 gene induced a decrease in fluorescence ranging from 90–95% compared with a siRNA control after incubation with 10 μM ND-DCP, suggesting that hNQO1 was responsible for the activation NQ-DCP to generate DCP with high fluorescence intensity (Figure 6B).

### 2.7. Fluorescence Imaging of hNQO1 in U87MG Tumor-Bearing Mice

**Figure 7:**
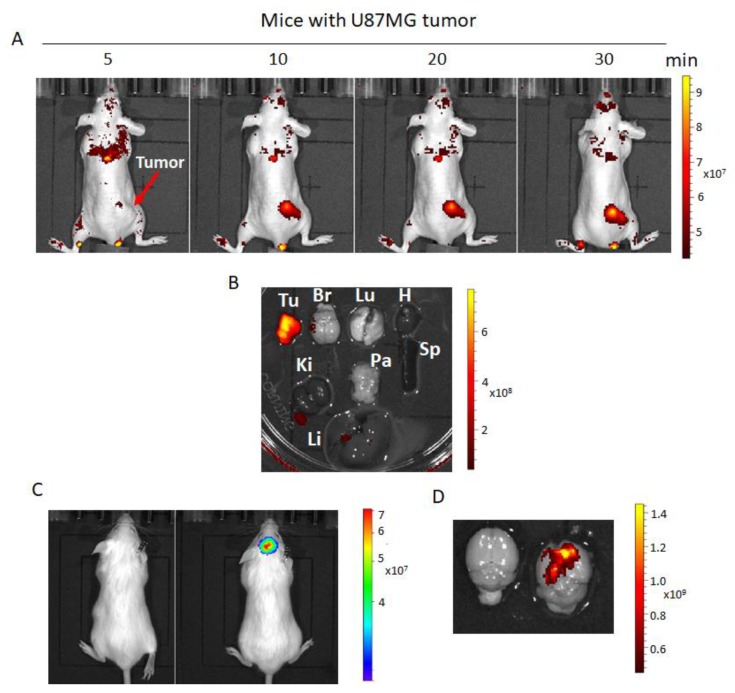
(**A**) In vivo real-time fluorescence imaging of endogenous NQO1 in subcutaneous U87MG tumor-bearing nude mice after intravenous administration of NQ-DCP (10 mg/kg, 50 µL). (**B**) Fluorescence imaging of ex vivo dissected organs namely, the spleen (Sp), Pancreas (Pa), Lungs (Lu), Brain (Br), Heart (H), Kidneys (Ki), and Liver (Li), along with tumor (Tu) after administration NQ-DCP at 30 min. (**C**) Luciferase-expressing U87MG cells were injected into the brains of NOD CRISPR Prkdc Il2r gamma (NCG) triple-Immunodeficient mice as described in materials and methods. Representative images of healthy and brain tumor-bearing mice after bioluminescence acquisition following luciferin administration. These images were acquired 15 days after intracranial implantation of U87MG cells. Images were acquired by setting the exposure time to ‘auto’ to limit the likelihood of an under or over-exposed image. The Living Image software program was used to analyze the tumors by drawing a region of interest (ROI) around each tumor in each image acquired during the bioluminescent imaging session. (**D**) Ex-vivo fluorescence imaging of healthy and tumor holding brain from mice after intracranial injection of NQ-DCP using excitation/emission filters 500/580. The scale indicates radiant efficiency.

With the prominent performance of NQ-DCP in cellular fluorescence imaging, we further applied this probe to evaluate its capacity for the noninvasive fluorescence imaging of hNQO1 in vivo. NQ-DCP (10 mg/kg, 50 μL) was intravenously injected into sub-cutaneous U87MG tumor-bearing mice. The whole-body fluorescence was imaged with a Caliper IVIS Spectral imaging system. As shown in Figure 7A, mice injected with NQ-DCP intravenously showed a gradual increase in fluorescence in the U87MG tumor, with the maximum fluorescence observed at 30 min. The host organs and tumor from the mice administered with NQ-DCP were harvested and their fluorescence was analyzed ex vivo using the IVIS to confirm the tumor-selective targeting ability of NQ-DCP. Fluorescence signals were selectively observed in the tumor and not in any other major organs such as the lung, heart, spleen, kidney, liver, brain, and pancreas (Figure 7B). The application of NQ-DCP to image hNQO1 activity was also investigated in intracranial U87MG tumor-bearing mice. Tumor xenografts were established by implanting exponentially growing U87MG-Luc cells by stereotaxic injection into mice brain and the tumor growth was assessed by quantitative bioluminescence using IVIS (in vivo imaging system) (Figure 7C). We were not able to observe any fluorescence signal by systemic injections of the probe in intracranial tumors; this is perhaps due to the inability of the NQ-DCP to cross the blood–brain barrier. Therefore, we performed intracranial injection of NQ-DCP (100 μM, 5 μL) to both healthy and tumor-bearing mice, the brain was isolated after 30 min and fluorescence images acquired. As shown in Figure 7D, a bright fluorescence signal observed from the tumor-bearing mouse brain and no fluorescence from the control healthy mouse brain.

## 3. Discussion

Development of hNQO1-activatable pro-fluorogenic probes that target the elevated hNQO1 found in solid tumors is useful for understanding enzymatic processes at the molecular level and developing tools to determine borders between diseased and healthy tissue during surgery. Consistently, based on the different levels of the hNQO1 present in cancer and normal cells, the catalytic property of NQO1 has been recently exploited for the development of effective fluorescent probes for cancer detection (Table 1) [23,24,25,26,27,28,29,30,31]. By exhibiting a small Stokes shift, the existing hNQO1 detecting fluorescent probes based on the rhodamine, fluorescein, cyanine, Nile red and BODIPY dyes reabsorb emitted photons, and lead to undesired background interferences. To overcome this problem, great efforts have been directed to develop fluorophore probes with a large Stokes shift (Δλ ≥ 80 nm). Here, we successfully developed a biocompatible, cell permeable and highly specific NQO1-responsive turn-on fluorescent probe (NQ-DCP; Figure 1A) with a large Stokes shift to detect cancer cells. In our design, Quinone propionic acid moiety was chosen as the enzyme active trigger and dicyanoisophorone group utilized as the chromophore due to its striking characteristics in terms of photophysical characteristics and biocompatibility.

The spectral character of NQ-DCP in response to hNQO1 was initially measured using both the UV−vis-absorption and fluorescence emission spectra upon the probe’s incubation with hNQO1 in PBS buffer in the presence of 100 μM NADH. The fluorescent spectra (Figure 2B) indicated that NQ-DCP was initially non-fluorescent and upon treated with hNQO1 had a significant increase in fluorescence intensity indicating the hNQO1 enzyme triggered cleavage reaction led to the release of highly fluorescent DCP (Figure 1B). The release of DCP in the presence of hNQO1 was further confirmed with HPLC analysis (Figure 1C). The fluorescence intensity of the probe was varied upon addition of different concentrations of hNQO1 (0−2.5 μg/mL) and the fluorescent signal intensities (Figure 2C) were linearly proportional to hNQO1 concentration (Figure 2D). Subsequently, the fluorescent intensity at 565 nm increased rapidly in a time-dependent manner and then obtained a maximum intensity in approximately 30 min (Figure 3A,B). To rule out possible activation of NQ-DCP by various enzymes and biomolecules present in mammalian cells, the fluorescence response of NQ-DCP solutions were exposed to GSH, ALDH, GGT, GPx, APE1, CBS, CTSL, NTR, and NADH alone or in combination of hNQO1. As Figure 3C,D illustrated, only hNQO1 could induce a remarkable fluorescence enhancement, whereas, other species exhibited a negligible fluorescence. Incubation of NQ-DCP with hNQO1 in the presence of its specific inhibitor, ES936 completely blocked the NQ-DCP fluorescence (Figure 3E).

Considering the excellent sensing properties of the probe in vitro system, we further studied the applicability of NQ-DCP to identify and differentiate tumor cells based on the presence of hNQO1 activity. Before cellular application, the cytotoxicity of the probe was evaluated in both normal and cancer cells and confirmed its biocompatibility (Figure 4A). As shown in Figure 4B, intense fluorescence was found in hNQO1 positive DAOY, T98G, and U87MG cells and no signals were observed from the hNQO1 negative normal cells. These observations indicated that the NQ-DCP is cell membrane permeable and the probe was readily activated by intracellular hNQO1 (Figure 4C). Simultaneously, the flow cytometry analysis showed that NQ-DCP has the ability to differentiate tumor cells based on hNQO1 activity in fluidic streams (Figure 5). The importance of hNQO1 for enzymatic activation the NQ-DCP probe was verified by pretreatment of cells with ES936, a selective inhibitor of hNQO1 and no fluorescence was found in hNQO1 inhibited cells after addition of NQ-DCP (Figure 6A). To further demonstrate the difference in fluorescence signal observed in the cell images is indeed caused by hNQO1 activity level, the fluorescence turn-on of NQ-DCP was evaluated in an hNQO1 knockdown T98G cell line. As shown in Figure 6B, limited or no fluorescence was observed after hNQO1 silencing. Having demonstrated successful detection of upregulated hNQO1 activity within various cancer cell lines, we explored the potential of NQ-DCP probe for in vivo identification of tumors in an animal model. As shown in Figure 7A, after the subcutaneous U87MG tumor-bearing mice were intravenously injected with NQ-DCP, a gradual increase in fluorescence response was observed in the tumor region of the mice in a time-dependent manner with a high tumor to background ratio. Ex vivo imaging of an alignment of individual mouse organs, demonstrating the presence of fluorescence signals only in the tumor and not in other selected major organs indicated the preferential accumulation and activation of NQ-DCP in the tumor tissue (Figure 7B). These observations are consistent with the reported preferential accumulation of fluorescent probes in tumor cells mediated by tumor hypoxia and an enhanced permeability retention (EPR), both characteristics of the aberrant tumor vasculature [41]. To better mimic the clinical scenario, we developed intracranial xenografts in nude mice by injecting U87MG cells (Figure 7C), and the probe was injected into the brain. The ex vivo fluorescence imaging (Figure 7D) showed that NQ-DCP was specifically activated by the tumor-bearing brain indicating the selective activation of the dye in the presence of hNQO1. These observations suggest that NQ-DCP could an effective fluorogenic probe for non-invasive and real-time imaging of hNQO1 activity in brain tumors during surgery.

## 4. Materials and Methods

### 4.1. Instruments and Materials

Fluorescence measurements were performed on a Hitachi F-2500 Fluorescence spectrophotometer (Hitachi-Science &Technology, Shizuoka, Japan) in a 10 mm standard cell with both excitation and emission slit widths of 10 nm. High-performance liquid chromatography (HPLC) was performed on Agilent HPLC instrument. IVIS Lumina XR Imaging system (Caliper Life Sciences, Inc., Waltham, MA, USA) was used for the in vivo imaging. The BD LSRFortessa™ cell analyzer (BD Biosciences, San Jose, CA, USA) was used for the flow cytometric analysis. 0.1% of BSA containing PBS with pH 7.4 was used for NQ-DCP spectroscopic measurements. The incubation of NQO1 with NQ-DCP in the presence of NADH was carried out on a shaker at 37 °C. Bio-rad ZOE fluorescent cell imager (Bio-Rad Laboratories, Hercules, CA, USA) was used for the fluorescent microscopy. All chemicals and solvents used in syntheses were purchased from Sigma- Aldrich (St. louis, MA, USA) or Fisher Scientific (Hampton, NH, USA) and used without further purification. NQO1 siRNA (h) (sc-37139) and NQO1 (A180) (sc-32793) antibodies were purchased from Santa Cruz (Santa Cruz Biotechnology Inc, Dallas, TX, USA). All cell lines were purchased from ATCC (American type cell culture collection). Immunodeficient NCG was purchased from Charles River Laboratories (Wilmington, MA, USA). Female athymic nude mice (nu/nu, 4–6 weeks) were purchased from Charles River Laboratories (Wilmington, MA, USA).

### 4.2. Spectroscopic Methods

For absorption and fluorescence measurements, all samples were first dissolved in 100% DMSO to obtain 10 mM stock solutions and then diluted to desired concentrations in PBS buffer containing 0.1% BSA for measurement. All spectroscopic measurements were performed under physiological conditions (pH 7.4 at room temperature. hNQO1 specific NQ-DCP activation was performed under philological conditions (PBS with 0.1% of BSA at 37 °C) in the presence of 100 μM NADH for 30 min. Time-dependent fluorescent intensity changes for different concentrations of NQ-DCI in the presence of NQO1 (2.0 μg/mL) and 100 μM NADH were measured using Hitachi F-2500 Fluorescence spectrophotometer (λ_ex_ = 420 nm and λ_em_ = 565 nm).

### 4.3. Selectivity Evaluation

To study the interference, NQ-DCP was incubated with various biologically relevant analytes such as thiols and other enzymes for 30 min with or without the addition of 100 μM NADH. Fluorescence spectra were analyzed to determine NQ-DCP specificity against hNQO1.

### 4.4. Evaluation of Inhibitor Efficiency

To evaluate the inhibitory efficiency of ES936 towards hNQO1 under in vitro conditions, different concentrations of ES936 (0–100 nM) incubated with 2.0 μg/mL of NQO1, 100 μM NADPH in PBS with 0.1% BSA (pH 7.4) at 37 °C. Fluorescence emission was recorded after 30 min of incubation (λ_ex_ = 420 nm).

### 4.5. Cell Culture, Fluorescence Imaging, and Flow Cytometry Analysis

Brain tumor cells and astrocytes were grown in Dulbecco’s modified Eagle’s (DMEM) medium containing 10% Fetal Bovine Serum and 1% penicillin/streptomycin in a humidified atmosphere of 5% CO_2_ at 37 °C. For fluorescence imaging, initially, cells were seeded into 6-well plate and cultured overnight in respective media at 37 °C. Media was replaced with fresh medium containing 10 μM NQ-DCP and incubated at 37 °C for 60 min and then the medium was replaced with PBS and live cells were imaged using a fluorescence microscope. For inhibitor study, cells were pretreated with NQO1 inhibitor ES936 (100 nM) for 6 h before adding the NQ-DCP. For determining endogenous hNQO1 activity in cancer cells using flow cytometry, about 10 × 10^4^ cells were incubated with 10 μM of NQ-DCP for 60 min and were analyzed using flow cytometry.

### 4.6. Knockdown of NQO1 by Small Interfering RNA (siRNA)

NQO1 siRNA (a pool of 3 target-specific 19-25 nt siRNAs, Santa Cruz Biotechnology, Inc., (Dallas, TX, USA) and scrambled siRNAs were introduced into cells using the X-tremeGENE siRNA transfection reagent (Roche, Basel, Switzerland) according to the manufacturer’s instructions with 80 pmol of NQO1 siRNA and 10 µl of transfection reagent per well of a six-well plate for T98G cells.

### 4.7. Immunoblotting

Cells were lysed in Cell Lysis Buffer (Cell Signaling, 9803) containing protease inhibitor and used for the immunoblotting. The protein concentration was estimated using the Bradford reagent (Bio-Rad, Hercules, CA, USA). Cell lysates with identical amounts of protein were fractionated by SDS-PAGE, and the proteins were electrophoretically-transferred to the polyvinylidene difluoride (PVDF) membranes. The hNQO1 and β-actin monoclonal antibodies and corresponding secondary antibodies were used for NQO1 protein detection.

### 4.8. In Vivo Bioimaging

The study was approved by the Institutional Animal Care and Use Committee (IACUC), Texas Tech University Health Sciences Center: Protocol No. 07050 entitled “Chemoprevention and Chemotherapy via MGMT”; Latest approval date: 28 July 2018, valid through 28 July 2019.

Four-week-old male and female NCG and athymic nude mice were housed in a micro ventilated caging system in a sterile environment and fed ad-libitum with standard irradiated research rodent diet and water. To detect endogenous NQO1 activity, NQ-DCP (10 mg/kg) was dissolved PEG-400: EtOH: saline (57.1:14.3:28.6, *v*/*v*/*v*) and 50 μL given through intravenous injection and animals were imaged using IVIS Lumina XR Imaging system. To establish U87MG xenografts, a total of 5 × 10^6^ cells (in 0.1 mL) were subcutaneously injected into the right inguinal area of the athymic nude mice. For establishing the orthotopic brain tumor model, the animals were anesthetized using 2% isoflurane and positioned in a Benchmark (Leica) stereotactic instrument [42]. A 27-gauge needle was then used to drill a burr hole into the skull 0.5-mm anterior and 2-mm lateral to the bregma. U87MG-Luc2 cell suspension (2 × 10^5^ cells in 5 μL PBS) was injected in the striatum at a depth of 5 mm from the dural surface over 10 min. These mice were imaged for bioluminescence five days post tumor inoculation and were observed for stable tumor growth for the next two weeks. To detect endogenous NQO1 activity, orthotopic brain tumor models, NQ-DCP (50 μM) was dissolved saline and 5 μL given through intracranial injection to both healthy and tumor-bearing mice. After 30 min of administration, brains were isolated and imaged using IVIS Lumina XR Imaging system.

## 5. Conclusions

In summary, we successfully developed an effective hNQO1 substrate- dicyanoisophorone (NQ-DCP) probe that was rapidly turned on in the presence of the cancer-specific biomarker, hNQO1, to yield a reporter whose spectral properties were sufficiently different from those of the probe so as to allow for ready differentiation of hNQO1 enzyme positive cancer cells from the negative normal cells. Combining its favorable light-up fluorescence feature, high selectivity, long wavelength emission, large Stokes shift, low cytotoxicity, and good membrane permeability, we applied NQ-DCP to achieve the real-time visualization of hNQO1 in live cells and mice. The characteristic target-to-background signal ratio (TBR) provided by the probe/reporter system allowed a facile microscopic detection and quantification cellular hNQO1 by flow cytometry. In addition, this fluorescent probe allowed a non-invasive visualization of hNQO1 in subcutaneous and in an orthotopic brain tumor-bearing mouse models. The data suggest that NQ-DCP could be an effective and promising fluorescent probe with applications in cancer bioimaging.

## Figures and Tables

**Table 1:** Fluorescent probes described for the human NAD(P)H quinone oxidoreductase-1 (hNQO1) detection and their comparison with new dicyanoisophorone (DCP) based fluorescent probe (NQ-DCP).

Fluorescent Probes for hNQO1 (Ref.)	λ_abs/em_ (Stokes-Shift)	Application	Fluorescent Probes for hNQO1 (Ref.)	λ_abs/em_ (Stokes-Shift)	Application
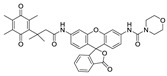 [25]	485/520 (35)	In vitro	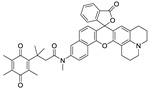 [26]	585/624 (39)	in vitro
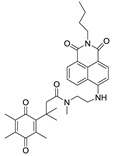 [28]	374/490 (116)	In vitro	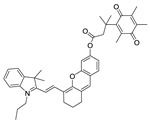 [35]	670/710 (40)	In vitro and in vivo
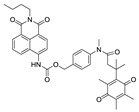 [27]	440/525 (85)	In vitro	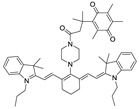 [35]	730/800 (70)	In vitro and in vivo
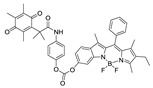 [31]	509/542 (33)	In vitro	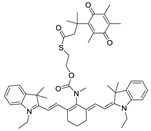 [34]	786/798 (12)	In vitro and in vivo
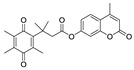 [30]	360/450 (90)	In vitro	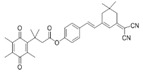 Present work	420/465 (145)	In vitro and in vivo

## Supplementary Materials

The following are available online at http://www.mdpi.com/2072-6694/10/12/470/s1, Scheme S1: synthetic scheme of NQ-DCP. Synthetic procedures of NQ-DCP along with intermediates and their ^1^H NMR, ^13^CNMR and Mass spectroscopic characterization. ^1^H NMR and ^13^CNMR spectra of NQ-DCP, QPA, DCP, and other intermediates.
