# Supplementary material for: Cancer-Specific Biomarker hNQO1-Activatable Fluorescent Probe for Imaging Cancer Cells In Vitro and In Vivo
Source: Cancers (Basel). 2018 Nov 28;10(12):470. doi: 10.3390/cancers10120470 (PMC6316840; doi:10.3390/cancers10120470)
Supplement: Supplementary file 1 [file cancers-10-00470-s001.pdf]

Supplementary Materials: Cancer-specific biomarker hNQO1 activatable fluorescent probe for imaging cancer cells in vitro and in vivo

Surendra Reddy Punganuru, Hanumantha Rao Madala, Viswanath Arutla and Kalkunte Srivenugopal

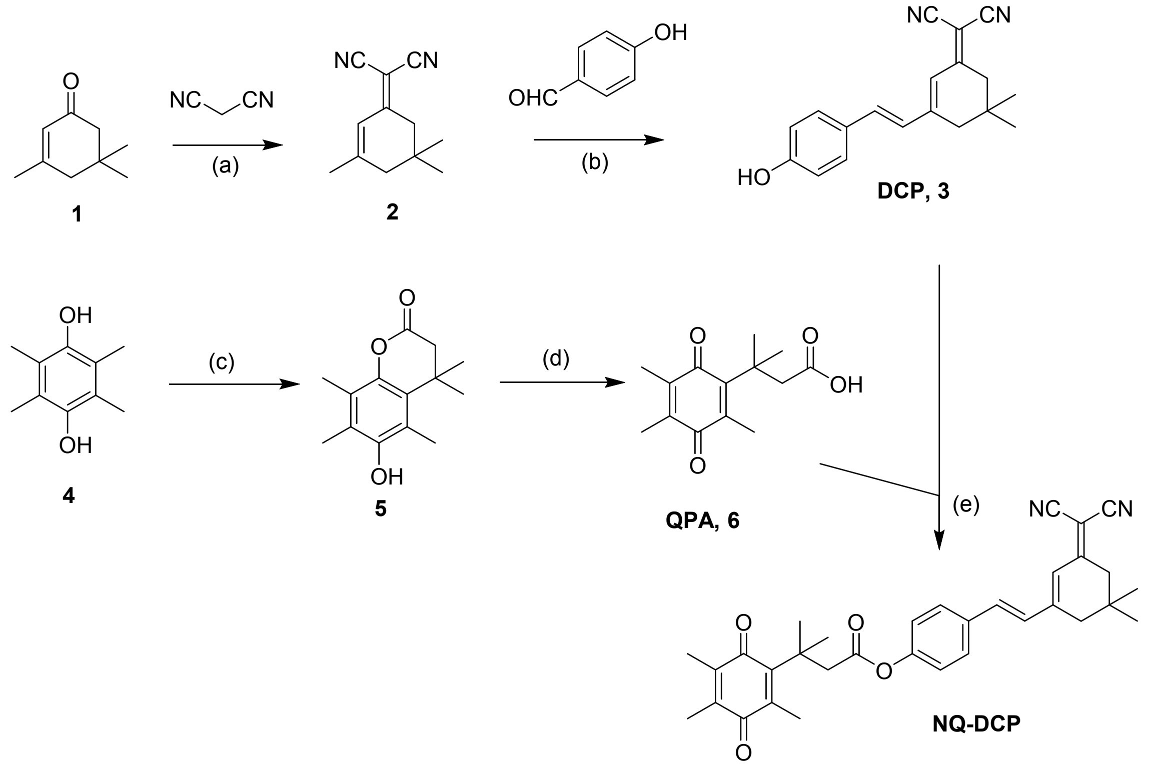

Scheme S1. Synthetic scheme of NQ-DCP. *Reagents & conditions:* (**a**) Ammonium acetate, acetic anhydride, acetic acid, toluene, reflux, 12 h; (**b**) piperidine, acetonitrile, 50 °C, 6 h; (**c**) methyl 3,3-dimethylacrylate, methane sulfonic acid, 70 °C, 2 h; (**d**) NBS, acetonitrile, water, rt, 1 h; (**e**) EDC, pyridine, rt, 10 h.

Synthesis of NQ-DCP

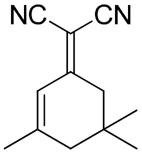

2-(3,5,5-trimethylcyclohex-2-en-1-ylidene) malononitrile (2): To a stirred solution of 2.25 mL (15.0 mmol) isophorone (**1**) and 1.49 g (22.5 mmol) of malononitrile in 50 mL toluene were added 0.29 g (3.75 mmol) of ammonium acetate, 0.5 mL of glacial acetic acid, and 1.0 mL of acetic anhydride. The reaction mixture was heated at 120 °C for 12 h under air atmosphere in the dark. Reaction mixture was allow to cool to room temperature, solvent was removed under diminished pressure, neutralized with saturated sodium carbonate solution and extracted with three 30 mL portion of ethyl acetate. The combined organic layer was dried over anhydrous Na_2CO_3, and concentrated. The crude product was purified by chromatography on a silica gel column. Elution with 9:1 hexanes-ethyl acetate afforded compound **2** as pale yellow solid: yield 1.98 g (71%); silica gel TLC R_f 0.37 (4:1 hexanes/ethyl acetate); ^{1}H NMR (400 MHz, $CDCl_3$) δ 1.01 (s, 6H), 2.03 (s, 3H), 2.18 (s, 2H), 2.51 (s, 2H) and 6.62 (s, 1H); ^{13}C NMR (100 MHz, $CDCl_3$) δ 25.3, 27.8, 32.4, 42.6, 45.6, 78.2, 112.4, 113.2, 120.5, 159.9 and 170.4.

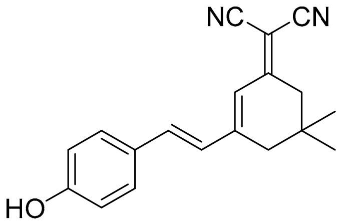

(*E*)-2-(3-(4-hydroxystyryl)-5,5-dimethylcyclohex-2-en-1-ylidene)malononitrile (DCP, 3): To a stirred solution of 1.92 g (10.3 mmol) of compound **2**, 0.15 mL of piperidine in 40 mL of acetonitrile was added 1.89 g (15.5 mmol) of 4-hydroxybenzaldehyde. The reaction mixture was stirred at 50 °C for 6 h. After the reaction mixture was allowed to cool to room temperature, the formed precipitate was filtered, washed with acetonitrile, and recrystallized with ethanol to obtained compound **3** as yellow crystal: yield 2.36 g (79%); ^{1}H NMR (400 MHz, $CDCl_3$) δ 1.08 (s, 6H), 2.46 (s, 2H), 2.59 (s, 2H), 6.81 (d, 1H, J = 14.8 Hz), 6.86 (d, 2H, J = 7.8 Hz), 7.02 (d, 1H, J = 16.0 Hz) and 7.42 (d, 2H, J = 8.0 Hz); ^{13}C NMR (100 MHz, $CDCl_3$) δ 28.0, 32.0, 39.2, 43.0, 77.7, 112.9, 113.7, 116.1, 122.8, 127.0, 128.6, 129.4, 136.8, 154.4, 157.2 and 169.5.

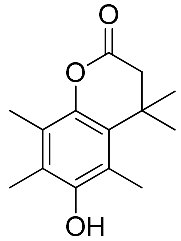

Synthesis of 6-hydroxy-4,4,5,7,8-pentamethylchroman-2-one (5): To a stirred solution of 2.25 g (13.5 mmol) of 2,3,5,6-tetramethylbenzene-1,4-diol (**4**) in 15 mL of methanesulfonic acid was added 1.98 mL (1.85 g, 16.2 mmol) of methyl 3,3-dimethylacrylate at room temperature. The reaction mixture was stirred at 70 °C for 2 h. After cooling to room temperature, the reaction mixture was diluted with 150 mL of water and extracted three 70 mL portions of dichloromethane. The combined organic extracts were washed with 100 mL of saturated $NaHCO_3$ solution, 100 mL of NaCl solution, dried over anhydrous Na_2SO_4 and concentrated under diminished pressure. The crude product was recrystallization from 30% $CHCl_3$ in hexanes gave compound **5** as white solid: yield 2.56 g (81%); ^{1}H NMR (400 MHz, $CDCl_3$) δ 1.45 (s, 6H), 2.19 (s, 3H), 2.22 (s, 3H), 2.36 (s, 3H), 2.55 (s, 2H) and 4.75 (s, 1H); ^{13}C NMR (100 MHz, $CDCl_3$) δ 12.3, 12.6, 14.5, 27.7, 35.5, 46.1, 118.9, 121.8, 123.4, 128.2, 143.5, 148.8 and 168.9.

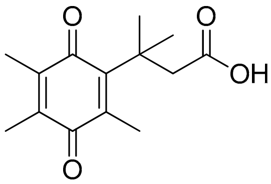

Synthesis of 3-methyl-3-(2,4,5-trimethyl-3,6-dioxocyclohexa-1,4-dien-1-yl)butanoic acid (QPA, 6): To a stirred solution of 2.00 g (8.54 mmol) of compound **5** in a solution of 70 mL of acetonitrile and 30 mL of water was added 1.67 g (9.39 mmol) of *N*-bromosuccinimide at room temperature. After 60 min, acetonitrile was removed from the reaction mixture, the residue was extracted three 30-mL portions of dichloromethane. The combined organic layer was dried over anhydrous Na_2SO_4 and concentrated under diminished pressure. The crude product was purified by chromatography on a silica gel column. Elution with 4:1 hexanes-ethyl acetate afforded compound **6** as yellow solid: yield 1.64 g (77%); silica gel TLC R_f 0.23 (7:3 hexanes/ethyl acetate); ^{1}H NMR (400 MHz, $CDCl_3$) δ 1.43 (s, 6H), 1.92 (s, 3H), 1.95 (s, 3H), 2.13 (s, 3H) and 3.01 (s, 2H); ^{13}C NMR (100 MHz, $CDCl_3$) δ 12.2, 12.6, 14.4, 29.0, 38.1, 47.4, 138.5, 139.2, 143.1, 152.1, 178.6, 187.6 and 191.0.

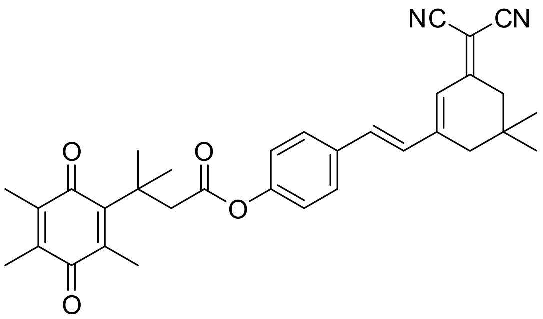

(*E*)-4-(2-(3-(dicyanomethylene)-5,5-dimethylcyclohex-1-en-1-yl)vinyl)phenyl 3-methyl-3-(2,4,5-trimethyl-3,6-dioxocyclohexa-1,4-dien-1-yl)butanoate (NQ-DCP, 7): To a stirred solution of 125 mg (0.50 mmol) of compound 6 in 1 mL of dry pyridine was added 193 mg (0.75 mmol) of EDC at room temperature. After 20 min a solution of 194 mg (0.50 mmol) of compound 3 in 0.2 mL of pyridine was added to the reaction mixture and stirred for 10 h at room temperature. The reaction mixture was diluted with 20 mL of 1N HCl and extracted with two 20 mL portions of ethyl acetate. Combined organic layer was dried over Na_2SO_4 and concentrated under diminished pressure. The obtained crude product was purified by chromatography on a silica gel column. Elution with 4:1 hexanes-ethyl acetate afforded compound 7 as yellow solid: yield 222 mg (85%); silica gel TLC R_f 0.22 (7:3 hexanes/ethyl acetate); ^{1}H NMR (400 MHz, $CDCl_3$) δ 1.08 (s, 6H), 1.53 (s, 6H), 1.93 (s, 3H), 1.94 (s, 3H), 2.18 (s, 3H), 2.46 (s, 2H), 2.60 (s, 2H), 3.26 (s, 2H), 6.83 (s, 1H), 6.92 (d, 1H, J = 16.1 Hz), 7.00 (s, 1H, J = 16.1 Hz), 7.03 (d, 2H, J = 7.8 Hz) and 7.49 (d, 2H, J = 8.6 Hz); ^{13}C NMR (100 MHz, $CDCl_3$) δ 12.1, 12.6, 14.4, 28.0, 28.9, 32.0, 38.4, 39.1, 42.9, 47.6, 78.8, 112.7, 113.4, 122.2, 123.7, 128.6, 129.3, 133.4, 135.7, 138.6, 139.3, 142.8, 151.3, 151.7, 153.6, 169.2, 171.1, 187.3 and 190.8.

1.H and 13C NMR spectrums of intermediates and NQ-DCP

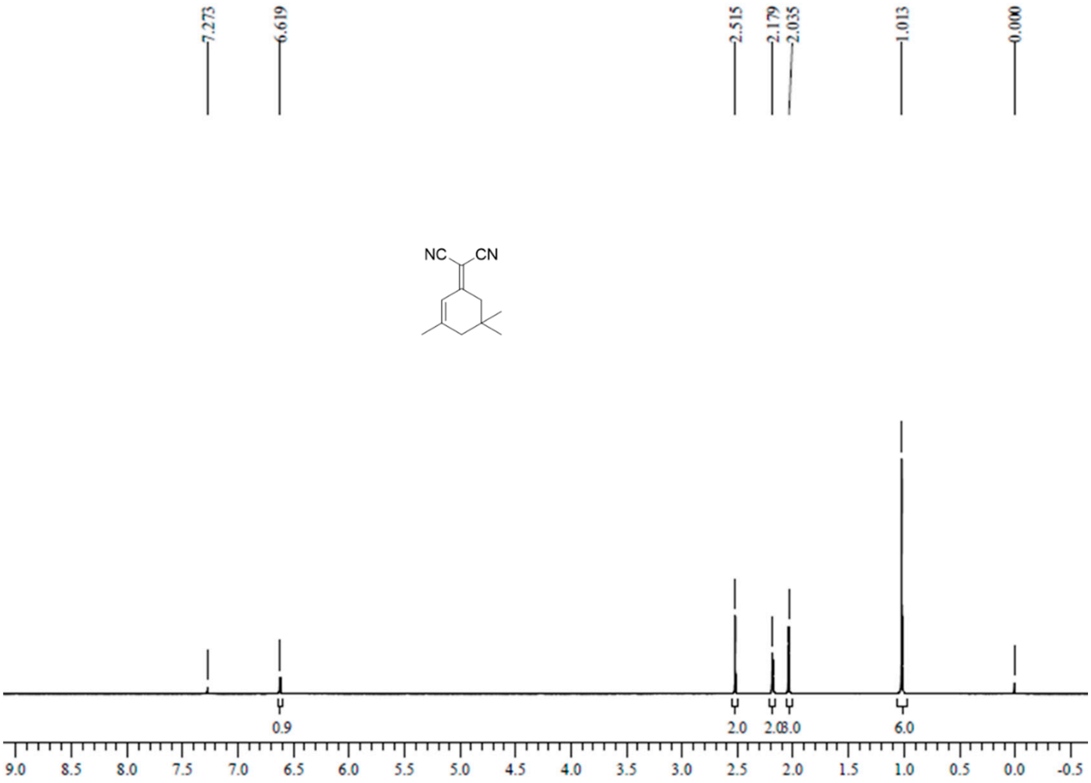

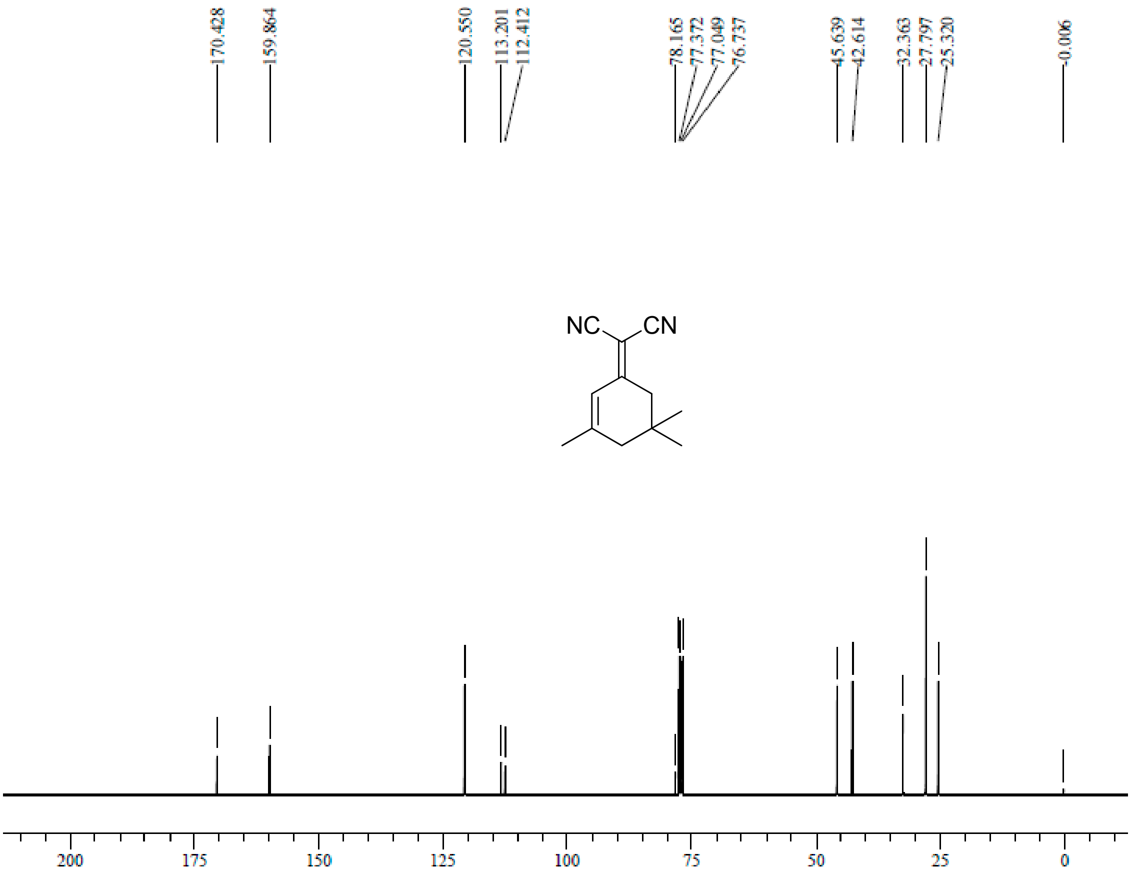
170.428
159.864
120.550
113.201
112.412
78.165
77.372
77.049
76.737
45.639
42.614
32.363
27.797
25.320
0.006
NC
CN
200
175
150
125
100
75
50
25
0

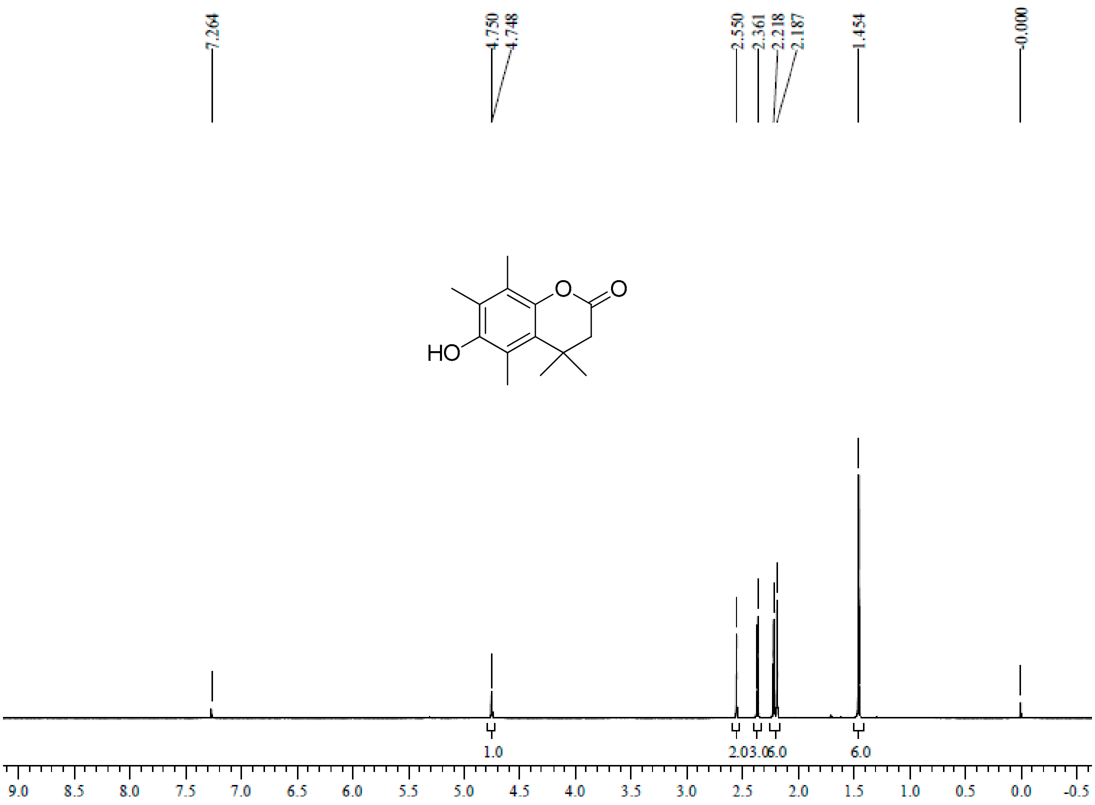
7.264
4.750
4.748
2.550
2.361
2.218
2.187
1.454
0.000
HO
O
O
1.0
2.0
3.0
6.0
6.0
9.0
8.5
8.0
7.5
7.0
6.5
6.0
5.5
5.0
4.5
4.0
3.5
3.0
2.5
2.0
1.5
1.0
0.5
0.0
-0.5

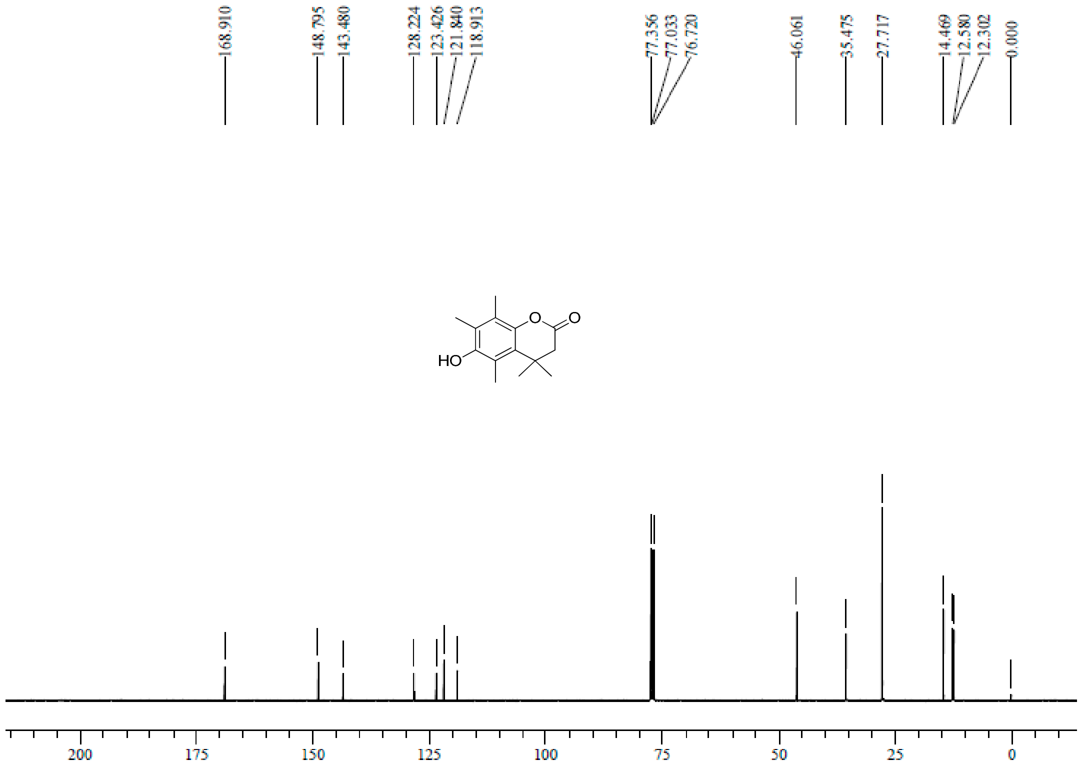
168.910
148.795
143.480
128.224
123.426
121.840
118.913
77.356
77.033
76.720
46.061
35.475
27.717
14.469
12.580
12.302
0.000
O
O
HO
200
175
150
125
100
75
50
25
0

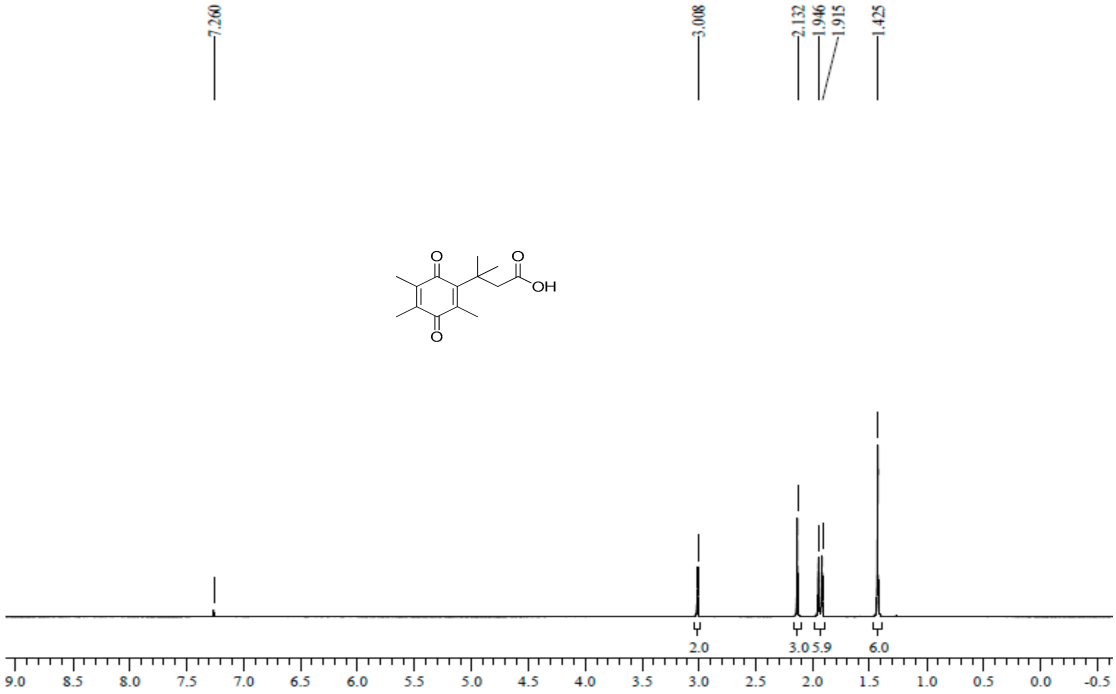
7.260
3.008
2.132
1.946
1.915
1.425
O
O
O
OH
2.0
3.0
5.9
6.0
9.0
8.5
8.0
7.5
7.0
6.5
6.0
5.5
5.0
4.5
4.0
3.5
3.0
2.5
2.0
1.5
1.0
0.5
0.0
-0.5

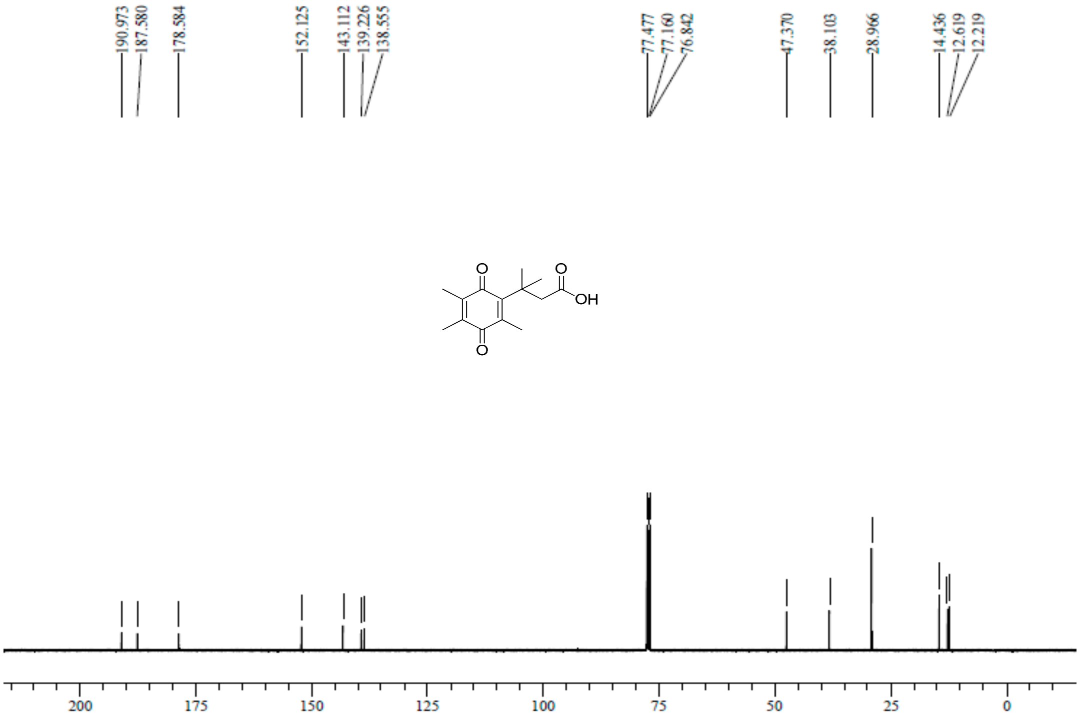
190.973
187.580
178.584
152.125
143.112
139.226
138.555
77.477
77.160
76.842
47.370
38.103
28.966
14.436
12.619
12.219
O
O
O
OH
200
175
150
125
100
75
50
25
0

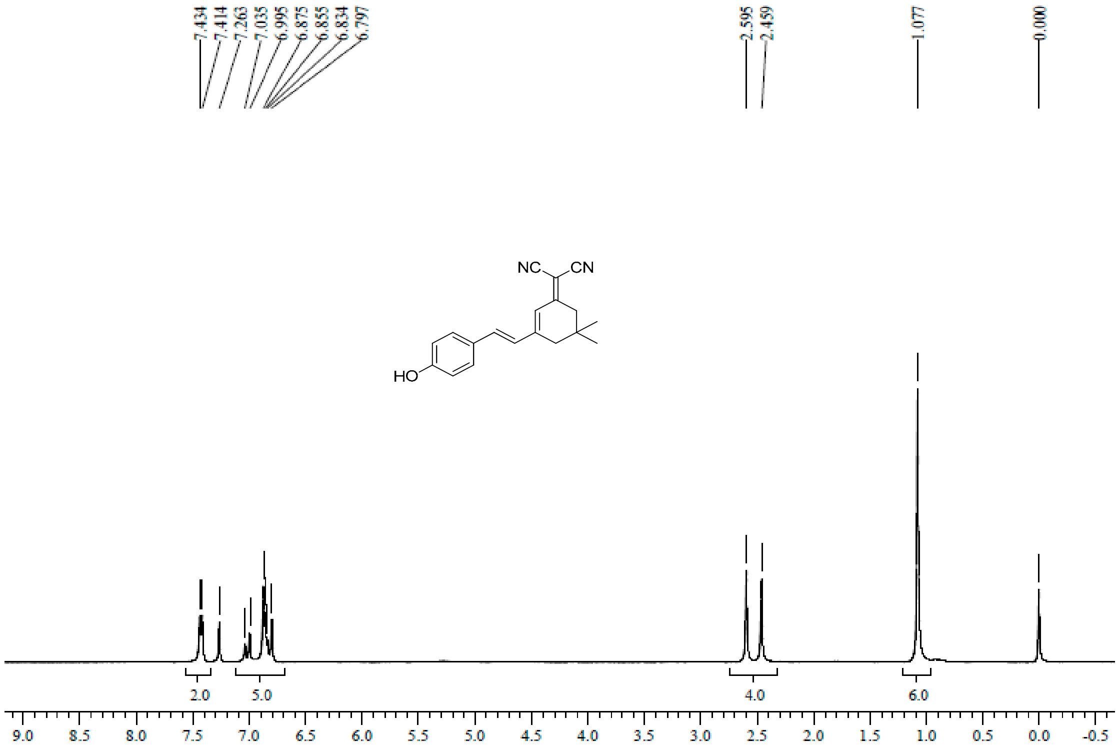
7.434
7.414
7.263
7.035
6.995
6.875
6.855
6.834
6.797
2.595
2.459
1.077
0.000
NC
CN
HO
2.0
5.0
4.0
6.0
9.0
8.5
8.0
7.5
7.0
6.5
6.0
5.5
5.0
4.5
4.0
3.5
3.0
2.5
2.0
1.5
1.0
0.5
0.0
-0.5

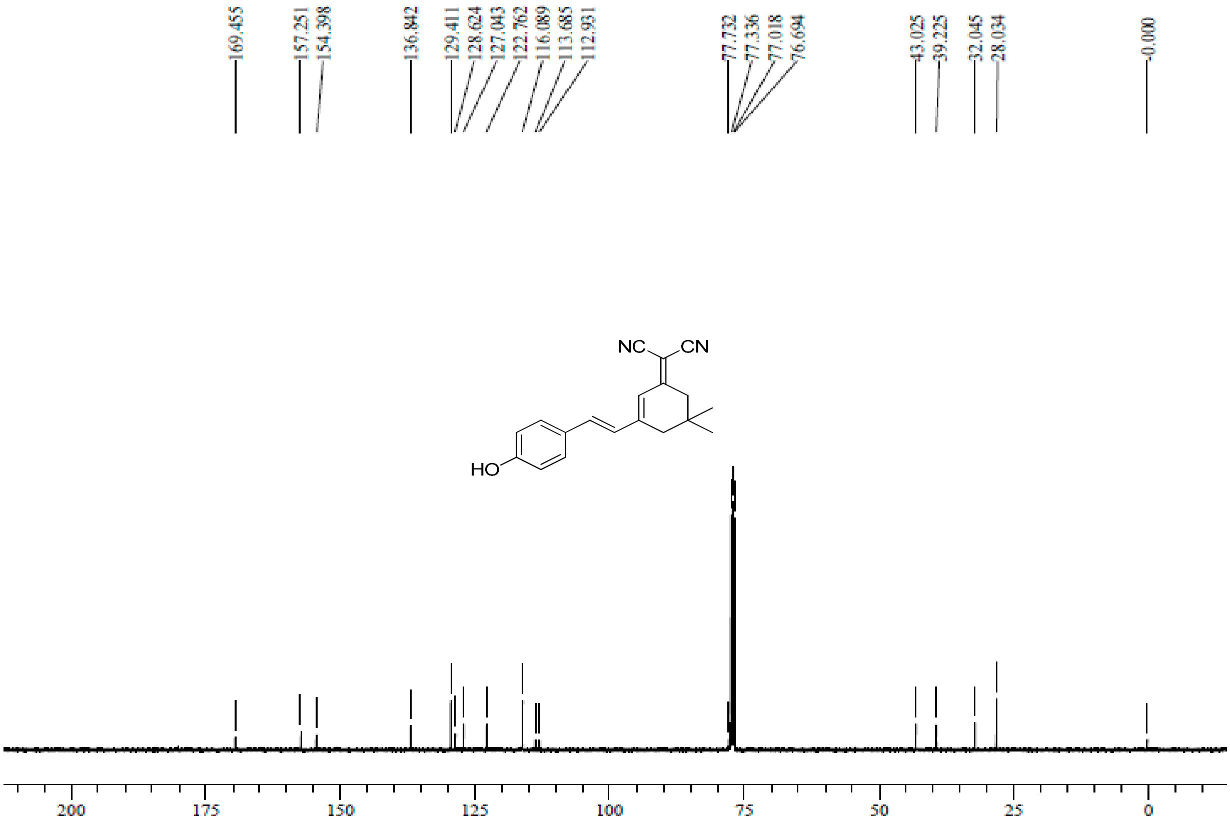
169.455
157.251
154.398
136.842
129.411
128.624
127.043
122.762
116.089
113.685
112.931
77.732
77.336
77.018
76.694
43.025
39.225
32.045
28.034
-0.000
NC
CN
HO
200
175
150
125
100
75
50
25
0

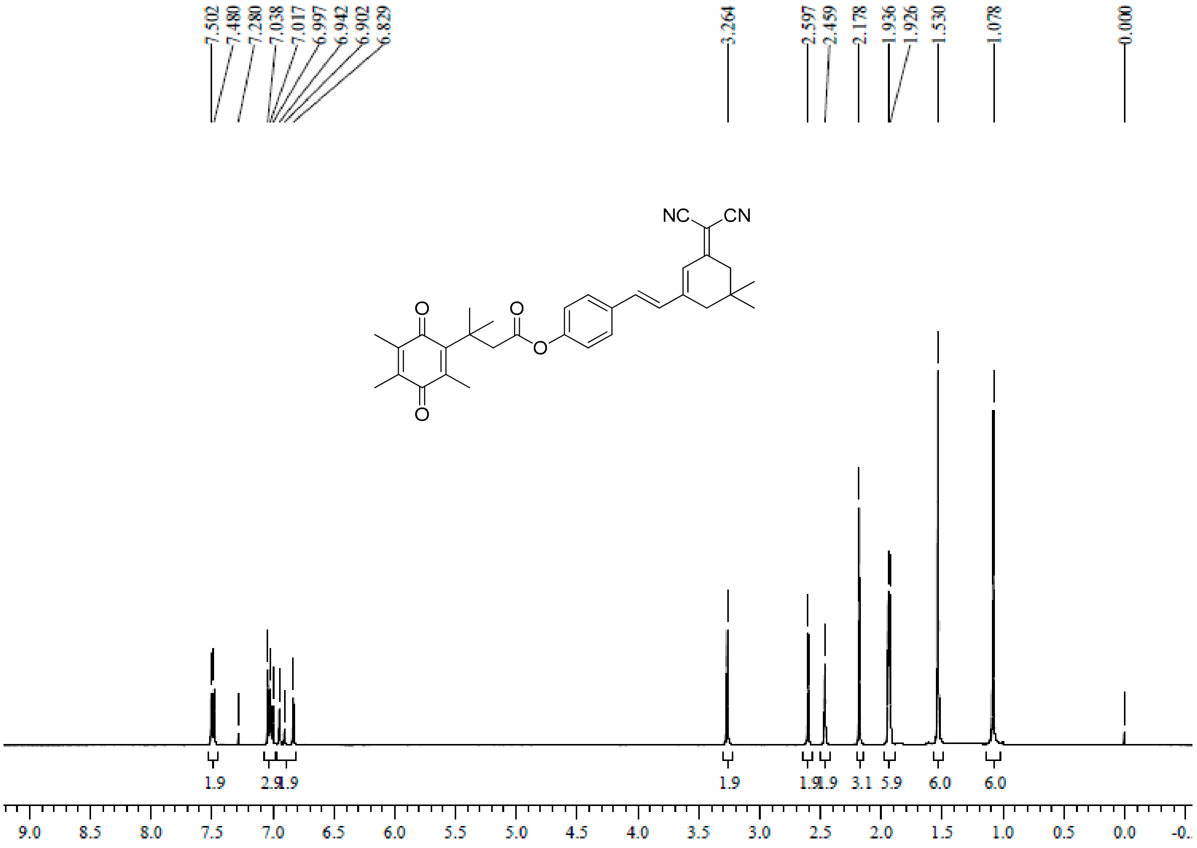
7.502
7.480
7.280
7.038
7.017
6.997
6.942
6.902
6.829
3.264
2.597
2.459
2.178
1.936
1.926
1.530
1.078
0.000
NC
CN
O
O
O
O
1.9
2.9
1.9
1.9
1.9
1.9
3.1
5.9
6.0
6.0
9.0
8.5
8.0
7.5
7.0
6.5
6.0
5.5
5.0
4.5
4.0
3.5
3.0
2.5
2.0
1.5
1.0
0.5
0.0

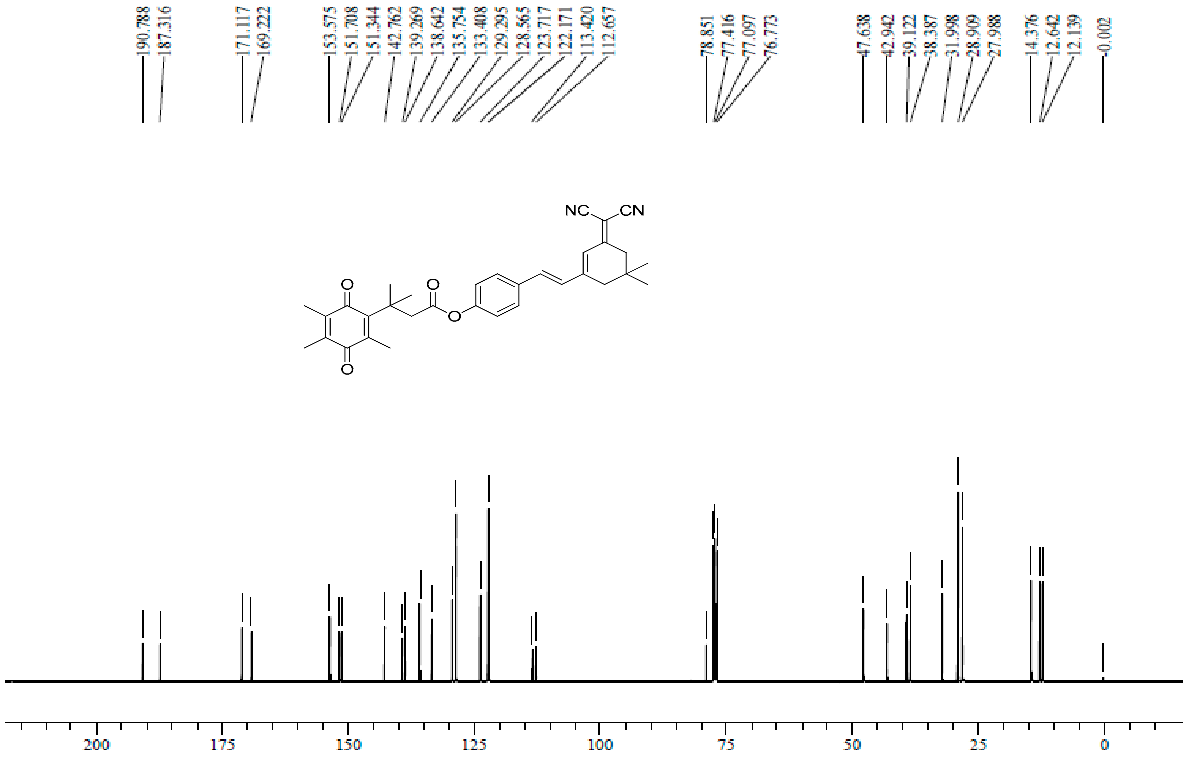
190.788
187.316
171.117
169.222
153.575
151.708
151.344
142.762
139.269
138.642
135.754
133.408
129.295
128.565
123.717
122.171
113.420
112.657
78.851
77.416
77.097
76.773
47.638
42.942
39.122
38.387
31.998
28.909
27.988
14.376
12.642
12.139
-0.002
NC
CN
O
O
O
O
200
175
150
125
100
75
50
25
0
